# Analysis of risk factors of axial neck pain in posterior cervical single-door laminoplasty from the perspective of cervical sagittal plane

**DOI:** 10.3389/fsurg.2022.973924

**Published:** 2022-09-14

**Authors:** Kang Kang Zuo, Wei Qin, Yu Miao, Lei Zhu

**Affiliations:** ^1^ Department of Orthopaedics, Renmin Hospital, Hubei University of Medicine, Shiyan, China; ^2^ Department of Orthopedics of Xiang Yang Central Hospital, Affiliated Hospital of Hubei University of Arts and Science, Xiang Yang, China; ^3^ Department of Orthopedics, Renmin Hospital of Yunyang District, Shiyan, China

**Keywords:** axial neck pain, T1 slope, logistic multivariate regression, sagittal vertex axis, cervical spondylotic myelopathy

## Abstract

**Objective:**

We carried out this study to explore the possible relationship between the cervical sagittal parameters in radiological images and axial neck pain (ANP) for patients who had underwent posterior cervical single-door laminoplasty.

**Method:**

141 patients were enrolled in the study from January 2018 to January 2021, among which 38 were enrolled into the ANP group and 103 were enrolled into the non-ANP group. C2–7 Cobb angle, C2–7 sagittal vertex axis (SVA), thoracic inlet angle, neck tilt, and T1 slope were measured using computed *tomography. Spearman correlation tests were used to analyze the possible correlation between radiological parameters and ANP.* Logistic regression was carried out to analyze the potential risk factor for the occurrence of ANP. Receiver operating characteristic (ROC) curve and area under the ROC curve were used to evaluate the significant result and the optimal diagnostic value.

**Results:**

As for radiographic parameters in the sagittal plane, the results suggested that only T1 slope and C2–7 SVA were statistically different between the ANP and non-ANP group (*p* = 0.001 and *p* = 0.047). Patients whose surgery involved the C2 spinous process demonstrated severe ANP symptoms than patients in the non-ANP group (*p* = 0.003). The Spearman correlation test showed that no statistical differences were found between visual analog scale (VAS) and radiological morphology parameters and only C2 involvement was found to correlate with postoperative VAS with respect to surgery. Logistic multivariate regression analysis demonstrated that only C2 involvement and T1 slope were significantly different when C2–7 SVA, T1 slope, C2 involvement together were included into consideration, with *p* values of 0.01 and 0.001.

**Conclusion:**

According to our research, C2 involvement and greater T1 slope were independent risk factors of ANP for the patients who underwent laminoplasty of cervical spine.

## Introduction

Cervical spondylotic myelopathy (CSM) could be described as a clinically symptomatic entity due to posterior or anterior compression of the spinal cord by degenerative diseases, while operation is necessary for patients suffering from CSM (1). The main purpose of the operation is to relieve the compression from the spinal cord. Traditional surgical approaches include anterior cervical decompression and fusion, cervical posterior single-door laminoplasty, or posterior laminectomy.

Cervical posterior laminoplasty was initially described for the management of cervical myelopathy resulting from multilevel stenosis secondary to ossification of the posterior longitudinal ligament, while the surgical method consisted of single-door and double-door laminoplasty (2). The five-lamina procedure (from C3 to C7) is the most popular cervical laminoplasty, but so far, no studies have been conducted on the number of laminae to be opened. Cervical posterior laminoplasty has its own indications, advantages, and complications. Common complications consist of C5 nerve palsy, axial neck pain (ANP), loss of lordosis, and loss of motion. However, Yukawa et al. confirmed that no substantial difference was detected between laminoplasty and laminectomy in terms of axial neck pain, cervical alignment, range of motion (ROM), and clinical outcomes (3).

**Figure 1:**
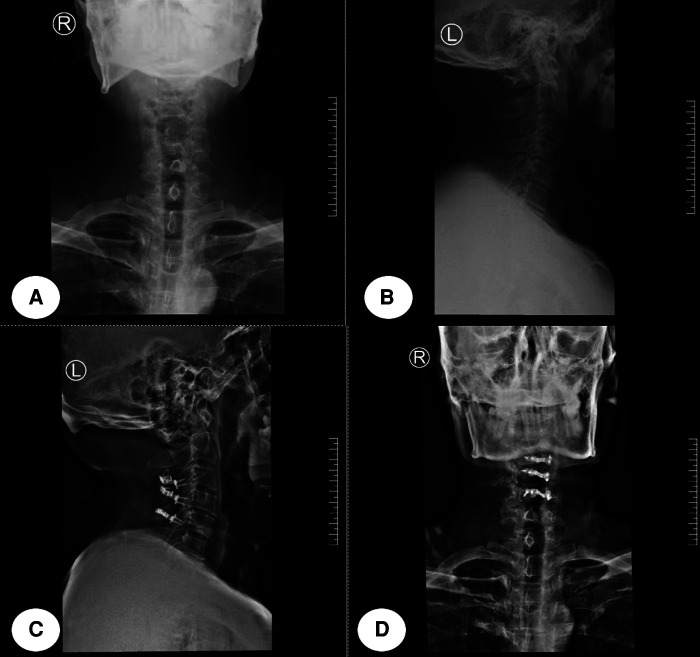
This was a typical case. A 45-year-old man was diagnosed with CSM. The VAS score was 1 before cervical posterior laminoplasty and the postoperative VAS turned to 4. [(**A**) and (**B**) showed the preoperative cervical x-rays both in anterior–posterior and lateral positions; (**C**) and (**D**) showed the postoperative cervical x-rays].

ANP could be a tricky clinical problem for a spine surgeon. Cervical spinal surgery was generally regarded less successful if patients suffered from postoperative ANP (4). ANP is also one of the most common complications after cervical laminoplasty (Figure 1). The symptoms of ANP were neck pain, stiffness, or dullness; however, predictors of persistent post laminoplasty neck pain still remained unclear. Kimura et al. carried out a study to reveal the possible predictors of persistent ANP after cervical laminoplasty, and the results showed that the presence of anterolisthesis was associated not only with the highest odds ratio of persistent ANP but also with significantly poorer functional outcomes (5). Sagittal imbalance of the cervical spine could be regarded as the main reasons for cervical disk degeneration and associated disorders. It was assumed that patients with cervical sagittal imbalance are more likely to develop ANP. A study carried out by Li et al. showed that greater T1 slope and larger C2–7 SVA might lead to the development of ANP when compared with the non-ANP group (6). However, there is no study on the prediction of ANP on sagittal parameters in patients with cervical posterior single-door laminoplasty.

## Method

### Patients

The patients enrolled were retrospectively studied from January 2018 to January 2021. The inclusion criteria were described as follows: (1) diagnosed with cervical spondylotic myelopathy or radiculopathy or myeloradiculopathy; (2) underwent posterior single-door laminoplasty surgery in our institution; (3) radiographical data were complete reserved including x-rays, MRI, and computed *tomography (*CT) scans. The exclusion criteria were as follows: (1) unclear diagnosis; (2) local infection, tumor, or other deformities; (3) previous cervical surgery; (4) unable to complete outpatient follow-up. At last, 141 patients were enrolled in this study.

### Surgical data

All of the patients had undergone posterior cervical single-door laminoplasty. The surgeries were conducted by the same group of surgeons followed by the same procedure. The decompression and fixation surgeries were described briefly as follows: The patients were in the prone position after anesthesia was performed with close monitoring. After the skin, subcutaneous, and fascia were cut, bilateral paraspinal muscles were peeled off to expose the posterior structure of vertebral. The surgery only cut the muscle longitudinally, not horizontally. At the same time, the muscles that did not interfere with the surgery were left intact. The posterior vertebral plates were turned over and then fixed in a position where the spinal canal was enlarged. Sometimes, the spinous process of C2 is too large for C3 vertebral to conduct a laminoplasty because it will restrain the displacement of the C3 vertebral plate. As a result, some part of the C2 spinous process is dissected and the end of cervical semispinalis and other small muscles were treated. Besides whether the C2 spinous process was treated, surgical information on the number of segments involved in surgery, operation time, and blood loss was then collected.

### Evaluation of ANP

ANP’s symptoms include neck pain, which is frequently complained, stiffness, and dullness. Visual analog scale (VAS) was used to evaluate ANP. Patients with a VAS score ≥3 were enrolled into the ANP group. Patients with a VAS score less than 3 were regarded as the non-ANP group. Pre- and postoperative VAS were then collected.

### Radiographical assessment

**Figure 2:**
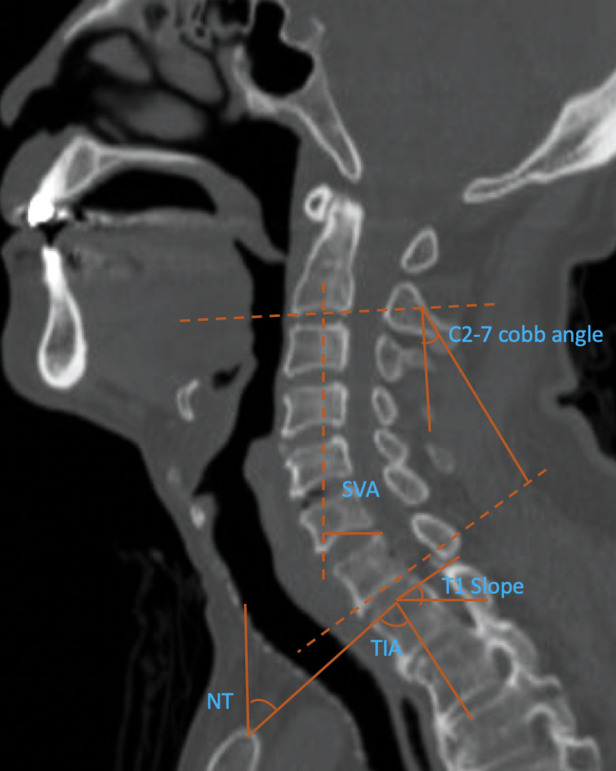
Radiographic evaluation of T1 slope, NT (neck tilt), and TIA (thoracic inlet angle), C2–7 Cobb angle, and C2–7 SVA (sagittal vertical axis).

As illustrated in Figure 2, we assessed all the radiographic parameters in CT scans. We surveyed into C2–7 SVA, C2–7 Cobb angle, thoracic inlet angle (TIA), neck tilt (NT), and T1 slope. C2–7 SVA was defined as the distance between the two vertical lines which crossed the center of C2 vertebral and the posterior superior corner of C7 vertebral. Cobb angle was defined as the angle of the two lines that crossed the inferior end plates of C2 vertebral and C7 vertebral. T1 slope was defined as the angle formed by the horizon line and the superior endplate of T1 vertebral. Neck tilt was defined as the angle formed by the vertical line and the line that crossed the cephalic end of the sternum and the center of the superior endplate of T1 vertebral. The line which was perpendicular to the superior endplate of T1 vertebral and the line connecting the cephalic end of the sternum and the center of the superior endplate of T1 vertebral formed the TIA.

### Statistics analysis

We used SPSS 22.0 (IBM, USA) for statistical analysis. Unpaired *t* test was used for continuous variables such as age, follow-up time, body mass index (BMI), blood loss in surgery, and radiographical parameters in the sagittal plane in the ANP and non-ANP groups. Categorical variables such as gender, smoking history, causes of disease, whether C2 were involved in surgery, and surgical side were calculated using the Chi-square test. To determine the correlation between VAS and the distinctive morphometric and surgical data, Kendall's tau-b and Spearman correlation were used. Multivariate logistic analyses were then conducted to determine the factors independently influencing the ANP occurrence. Furthermore, to acquire the most effective parameters that could predict the occurrence of ANP, ROC (the area under the receiver operating characteristics curve, AUC) was used. A *p* value less than 0.05 was considered statistically different.

## Results

### Characteristics of the enrolled patients and surgical data

There were 141 patients enrolled in our study, among which 38 were enrolled into the ANP group and 103 were enrolled into the non-ANP group at the final follow-up. As for the 38 patients in the ANP group, 10 patients developed ANP within 1 month after surgery, while the symptoms aggregated and persisted for 1 year without resolution. The other 28 patients developed ANP after 1 month postoperatively and the symptoms were still present at the last follow-up. No statistical difference was noticed (*p* > 0.05) in baseline information including age, gender, follow-up time, BMI, smoking history, or causes of disease (Table 1). When it comes to surgical data, surgical side, number of segments involved, operation time, and blood loss in surgeries showed no statistical difference, while the surgical procedure was distinctly different in the two groups (Table 2). Patients whose surgery involved the C2 spinous process demonstrated severe ANP symptoms than the non-ANP group (*p* = 0.003).

**Table 1:** Characteristics of the enrolled groups treated with single-door laminoplasty.

Characteristics		ANP group	Non-ANP group	χ^2^ or *t* value	*p* value
Age (years)		57.1 ± 10.1	55.5 ± 8.7	0.915	0.362
Gender	Male	25	59		
	Female	13	44	0.834	0.361
Follow-up time (months)		23.3 ± 2.7	23.9 ± 4.2	0.760	0.448
BMI (kg/m^2^)		22.9 ± 2.4	22.6 ± 2.8	0.628	0.531
Smoking history	Yes	15	33		
	No	23	70	0.683	0.408
Causes of disease	Myelopathy	28	76		
	Radiculopathy	4	9		
	Myeloradiculopathy	6	18	0.143	0.931

ANP, axial neck pain; BMI, body mass index.

**Table 2:** Surgical information of the enrolled groups treated with single-door laminoplasty.

Characteristics		ANP group	Non-ANP group	χ^2^ or *t* value	*p* value
C2 involvement	Yes	20	27		
	No	18	76	8.718	0.003*
Surgical side	Left	17	54		
	Right	21	49	0.657	0.418
Number of segments	3	10	25		
	4	12	48		
	5	16	30	2.950	0.229
Operation time		125 ± 30	118 ± 27	2.432	0.874
Blood loss (ml)		107.2 ± 23.5	101.1 ± 19.4	1.567	0.120

*p < 0.05.

ANP, axial neck pain.

### Comparison of pre- and postoperative VAS in the ANP and non-ANP groups

We then inspected the VAS score before operation in the two groups. No statistical difference was found with a *p* value of 0.598. Compared with the preoperative VAS score, patients in the ANP group demonstrated a distinct rise of postoperative VAS. Among all the patients in the non-ANP group, 37 patients had improvement. 38 had equal pain and 28 got worse (Table 3).

**Table 3:** Pre- and postoperative VAS and the change of VAS after surgery in two groups.

Characteristics		ANP group	Non-ANP group	χ^2^ or *t* value	*p* value
VAS before surgery		0.95 ± 0.80	0.86 ± 0.84	0.528	0.598
VAS after surgery		3.79 ± 0.90	0.90 ± 0.82	17.9	<0.01
VAS change	Worsen	38	28		
	Equal	0	38		
	Improved	0	37	—	<0.001

VAS, visual analog scale.

### Radiographic assessment at the sagittal plane

As for radiographic parameters in the sagittal plane, the results suggested that only the T1 slope and C2–7 SVA were statistically different in the ANP group and the non-ANP group (*p* = 0.001 and *p* = 0.047) (Table 4). C2–7 SVA was 16.7 ± 6.2 in the ANP group, which was higher than that of the non-ANP group, which is 14.5 ± 1.5. T1 slope was 26.2 ± 5.6 in the ANP group, which is also higher than that of the non-ANP group, which is 22.0 ± 6.7. Other parameters showed no statistically differences in both groups (*p* > 0.05).

**Table 4:** Radiographic measurement of all the patients preoperatively at the sagittal plane.

Characteristics	ANP group	Non-ANP group	χ^2^ or *t* value	*p* value
C2–7 SVA	16.7 ± 6.2	14.5 ± 5.5	2.003	0.047*
Cobb angle (°)	12.3 center6.8	13.7 center9.6	0.869	0.386
TIA (°)	72.5 center8.6	69.9 center11.1	1.286	0.201
T1 slope (°)	26.2 center5.6	22.0 center6.7	3.366	0.001*
Neck tilt (°)	46.3 center6.9	47.9 center8.0	1.06	0.291

*p < 0.05.

ANP, axial neck pain; SVA, sagittal vertex axis; TIA, thoracic inlet angle.

### Correlation between VAS and preoperative morphometric and surgical data

**Figure 3:**
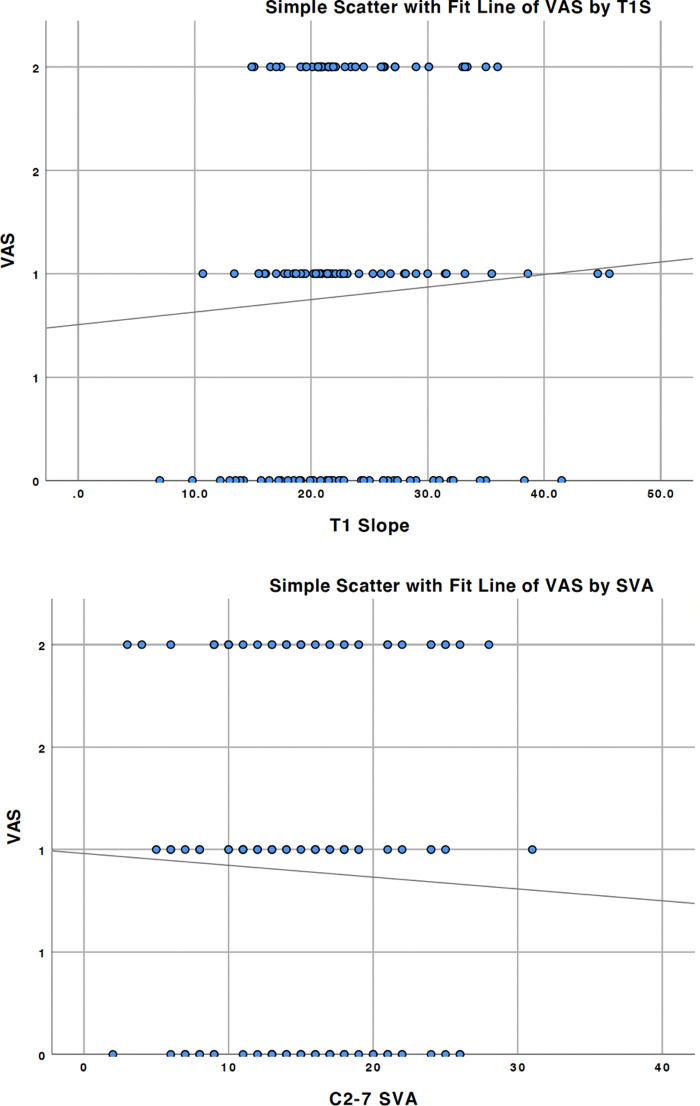
Simple scatter with fit line of preoperative VAS by T1 slope and C2–7 SVA, with a *p* value of 0.510 and 0.467, respectively.

After knowing preoperative T1 slope, C2–7 SVA and C2 involvement showed statistical differences in the comparison between two groups in radiological and surgical data. We then used the Spearman correlation test to determine whether these variables were related to VAS. The results showed that the T1 slope and C2–7 SVA showed no statistical difference, with a *p* value of 0.510 and 0.467, respectively (Table 5). Figure 3 showed the simple scatter with fit line of preoperative VAS by T1 slope and C2–7 SVA. However, Kendall's tau-b test of C2 involvement (surgical data) and postoperative VAS showed a *p* value less than 0.05, which is 0.043.

**Table 5:** Correlation between VAS and morphometric and surgical data.

		Correlation coefficient	*p*
Before surgery	VAS and T1 slope	0.056	0.51
	VAs and C2–7 SVA	−0.062	0.467
After surgery	VAS and C2 involvement	0.153	0.043*

*p < 0.05.

VAS, visual analog scale.

### The correlation between VAS and postoperative radiological data

**Figure 4:**
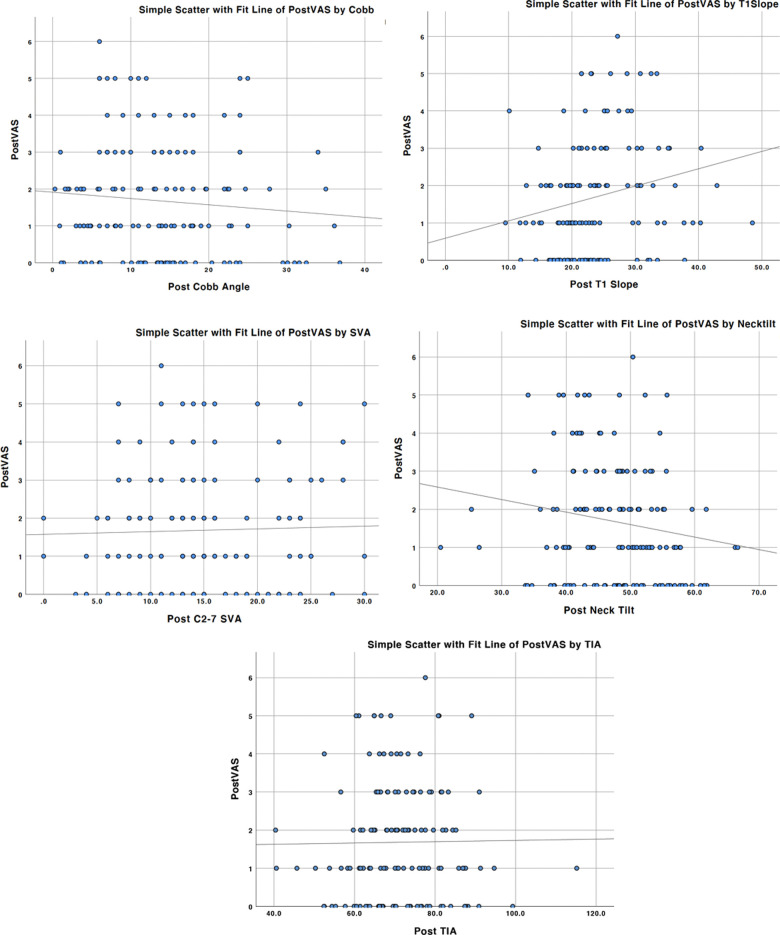
Simple scatter with fit line of postoperative VAS by Cobb angle, T1 slope, C2–7 SVA, neck tilt, and TIA, with *p* values all more than 0.05.

T tests were applied to detect whether there was diffidence between pre- and postoperative radiological parameters. All the variables in sagittal plane showed no differences pre- and postoperatively in our study (Table 6). We further analyzed the correlation between VAS and postoperative radiological parameters. Figure 4 showed the simple scatter with fit line of postoperative VAS by radiological parameters. No obvious linear relationship was found in the Spearman correlation test.

**Table 6:** Radiographic measurement of all the patients pre- and postoperatively at the sagittal plane.

Characteristics	Before surgery	After surgery	χ^2^ or *t* value	*p* value
C2–7 SVA	15.1 center5.8	14.8 center6.5	0.33	0.742
Cobb angle (°)	13.3 center8.9	13.8 center8.4	0.629	0.484
TIA (°)	70.5 center10.6	71.7 center11.1	0.344	0.731
T1 slope (°)	23.1 center6.7	23.5 center6.9	0.419	0.678
Neck tilt (°)	47.4 center7.8	48.2 center7.9	0.113	0.910

SVA, sagittal vertex axis; TIA, thoracic inlet angle.

### Logistic regression analysis

To discover the risk variables that were closely related to ANP symptoms, we conducted a multivariate regression analysis, and the results of the logistic multivariate regression analysis demonstrated that only C2 involvement and T1 slope were significantly different when C2–7 SVA, T1 slope, C2 involvement together were included into consideration, with *p* values of 0.01 and 0.001 (Table 7). When a surgery was involved in C2, the risk of ANP occurrence turned to 2.959-fold higher than that with an intact C2 structure.

**Table 7:** Logistic regression of risk factors for ANP.

Risk factors	*p* value	Odds ratio (95% confidence interval)
C2–7 SVA	0.096	1.062 (0.989–1.141)
T1 slope	0.001*	1.103 (1.038–1.172)
C2 involvement	0.01*	2.959 (1.295–6.760)

*p < 0.05.

ANP, axial neck pain; SVA, sagittal vertex axis.

### ROC curve analysis and prediction threshold

**Figure 5:**
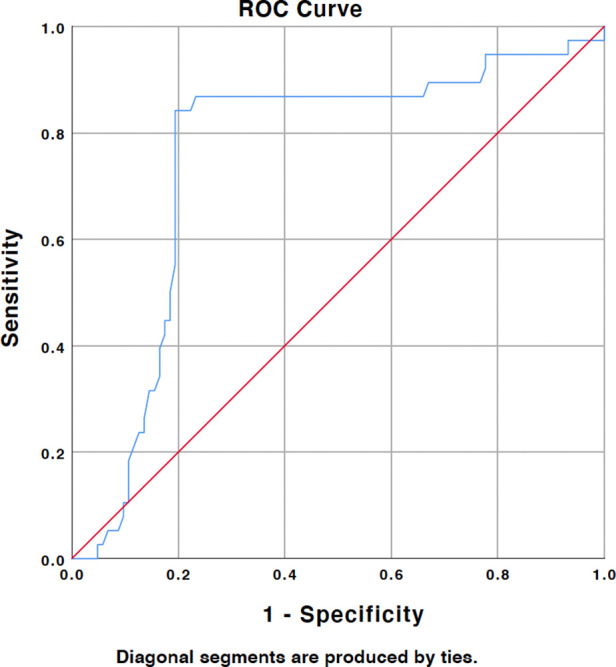
ROC analysis demonstrated that the cutoff value of T1 slope is 23.1° and AUC (area under the curve) was 0.752.

ROC curve suggested that the cut off value for the T1 slope was 23.1° at which the model acquired a higher Youden index. When regarding 23.1° as a prediction threshold, the area under the characteristics curve (ROC) was 0.752, which demonstrated a good prediction value (Figure 5).

## Discussion

Cervical sagittal alignment has been considered to be important for the growth and degeneration of the whole spine. Studies showed that imbalanced cervical spine was more liable to cause cervical disk degeneration when compared with the balanced subjects (7, 8). Some other studies have elucidated the link between sagittal alignment and health related quality of life (HRQOL) outcomes, and the results confirmed that increasing sagittal imbalance as defined by the sagittal vertical axis (SVA) has been strongly correlated with HRQOL (9–11). Recognizing the normal variations in cervical spine sagittal profile also helps determine the optimal cervical spine alignment for cervical spine fusion, since it could minimize complications after the surgery. Cervical sagittal alignment could also be recognized as the risk factor for the complications of anterior cervical surgery, such as adjacent segment diseases (ASD). A study carried out in 2018 concluded that T1 slope of less than 19.50 appears to be an independent risk factor for the ASD, keeping the T1 slope of more than 19.5 reasonable to avoid the occurrence of ASD after the anterior cervical surgery (12). For the relation between posterior cervical surgery and cervical sagittal alignment, Kennamer et al. carried out a study in 2019 to examine the dataset of all patients adopting posterior cervical decompression and fusion, and the results showed that poor cervical alignment predicts poorer clinical outcomes as well as need for revision (13). Xu et al. examined the relationship between preoperative cervical sagittal parameters and clinical outcome in patients with posterior longitudinal ligament ossification treated by laminoplasty, and subjects who had high center of gravity of the head-C7 SVA levels prior to surgery may develop sagittal imbalances and neurological symptoms. (14).

The pathogeny of ANP is widely considered to be multifactorial, while ANP after the laminoplasty was usually considered to be an important factor in the choice of alternative procedure. Some studies stated that facet disruption or muscle dissection may lead to the occurrence of ANP. Hosono et al. tried to compare the incidence of ANP after the laminoplasty and anterior fusion, and the results demonstrated that the incidence of ANP was statistically higher in the patients who underwent laminoplasty than in the patients who underwent anterior fusion (60% vs. 19%, respectively) (15). Another study carried out by Hosono et al. enrolled 37 patients adopting laminoplasty from C3 through C7, in which 31 patients were left-sided muscle dissection, 23 patients were right-sided muscle dissection (31 vs. 23, from C3 through C6), and the results confirmed that 49% of the patients suffered from ANP after the laminoplasty and 15% of patients in the left- or right-sided dissection C3 through C7 laminoplasty groups, with the conclusion that C7 preservation was a more important factor with regard to the occurrence of ANP (16). Some surgeons prefer C3 laminectomy rather than laminoplasty, as C3 laminectomy does not require full exposure of the C3 lamina and lessens disruption of the extensor muscle insertion at C2, as the researcher believes that too much excision of C2 muscles can lead to the occurrence (2). In the study, the results showed that the risk of ANP occurrence turned to 2.959-fold higher than that with an intact C2 structure, when the laminoplasty was involved in C2, while the conclusion is similar to previous studies. The T1 slope is considered to be the only value that links both the cervical and thoracic spine, which shows a close correlation with thoracic kyphosis, TIA (17, 18). Some other study confirmed that higher thoracic kyphosis often results in a greater T1 slope in most cases (19, 20). Li et al. carried out a study to explore the relation between T1 slope and the occurrence of ANP, and the results showed that patients require strengthening of the posterior paraspinal neck muscles, especially patients with a greater T1 slope so as to minimize the energy expenditure, while the ANP patients usually correspond to larger T1 slope (6). For patients with laminoplasty, greater T1 slope and C2 involvement during the surgery may lead to the occurrence of ANP. In these patients with a large T1 slope, greater muscle strength is often required to maintain normal physiological curvature and spinal balance. Since posterior laminoplasty itself causes damage to the muscles, postoperative muscle strength is not sufficient to maintain sagittal balance, further leading to ANP.

Several surgical techniques have been proposed to reduce the incidence of complications after laminoplasty. Signorelli et al. conducted a single-center investigation on the outcome of the revised cervical laminoplasty, which consisted of bilateral exposure, spinous process removal, and symmetrical muscular closure (21). The result showed that applying some few variations to a standard monoliteral approach remarkably improved the clinical outcomes. At the same time, as stated above, Kudo et al. concluded that cervical laminoplasty combined with C3 laminectomy decreased postoperative ANP (22). In addition, some other researchers hold the preservation of the C7 spinous process and the attachment of nuchal muscles could also reduce the incidence of ANP (23).

Some limitations of the study still exist in the study. First, global spinal sagittal radiographs should be evaluated so as to estimate the mutual effect of the lumbar and thoracic spine. Second, this is a retrospective study, and a prospective study or randomized controlled study is still needed to further evaluate the relation between the cervical sagittal parameters and the occurrence of ANP. Third, the number of patients included in the study is too small, and more patients should be included in further research.

## Conclusions

We found that C2 involvement and greater T1 slope were the independent risk factors of ANP for the patients adopting single-door laminoplasty of cervical spine.

## Data Availability

The original contributions presented in the study are included in the article/Supplementary Material, further inquiries can be directed to the corresponding author/s.
